# Correction: Wang, M., et al. HSP70–eIF4G Interaction Promotes Protein Synthesis and Cell Proliferation in Hepatocellular Carcinoma. *Cancers* 2020, *12*, 2262

**DOI:** 10.3390/cancers12113410

**Published:** 2020-11-18

**Authors:** Meng Wang, Kai Wei, Baifeng Qian, Svenja Feiler, Anastasia Lemekhova, Markus W. Büchler, Katrin Hoffmann

**Affiliations:** Department of General, Visceral, and Transplantation Surgery, Ruprecht Karls University, Im Neuenheimer Feld 110, 69120 Heidelberg, Germany; tf225@stud.uni-heidelberg.de (M.W.); k.wei@stud.uni-heidelberg.de (K.W.); qianbf@mail.sysu.edu.cn (B.Q.); Svenja.Feiler@gmx.net (S.F.); Anastasia.Lemekhova@med.uni-heidelberg.de (A.L.); markus.buechler@med.uni-heidelberg.de (M.W.B.)

The authors wish to make the following corrections to this paper [1]:

There was a mistake in the original version of the article in Figure 1. The label of “Tumor” and “Non-tumor” in Figure 1A is reversed. This is due to a mistake in the figure editing. The figure legend does not need to be changed.

Thus, the original Figure 1 listed below: 
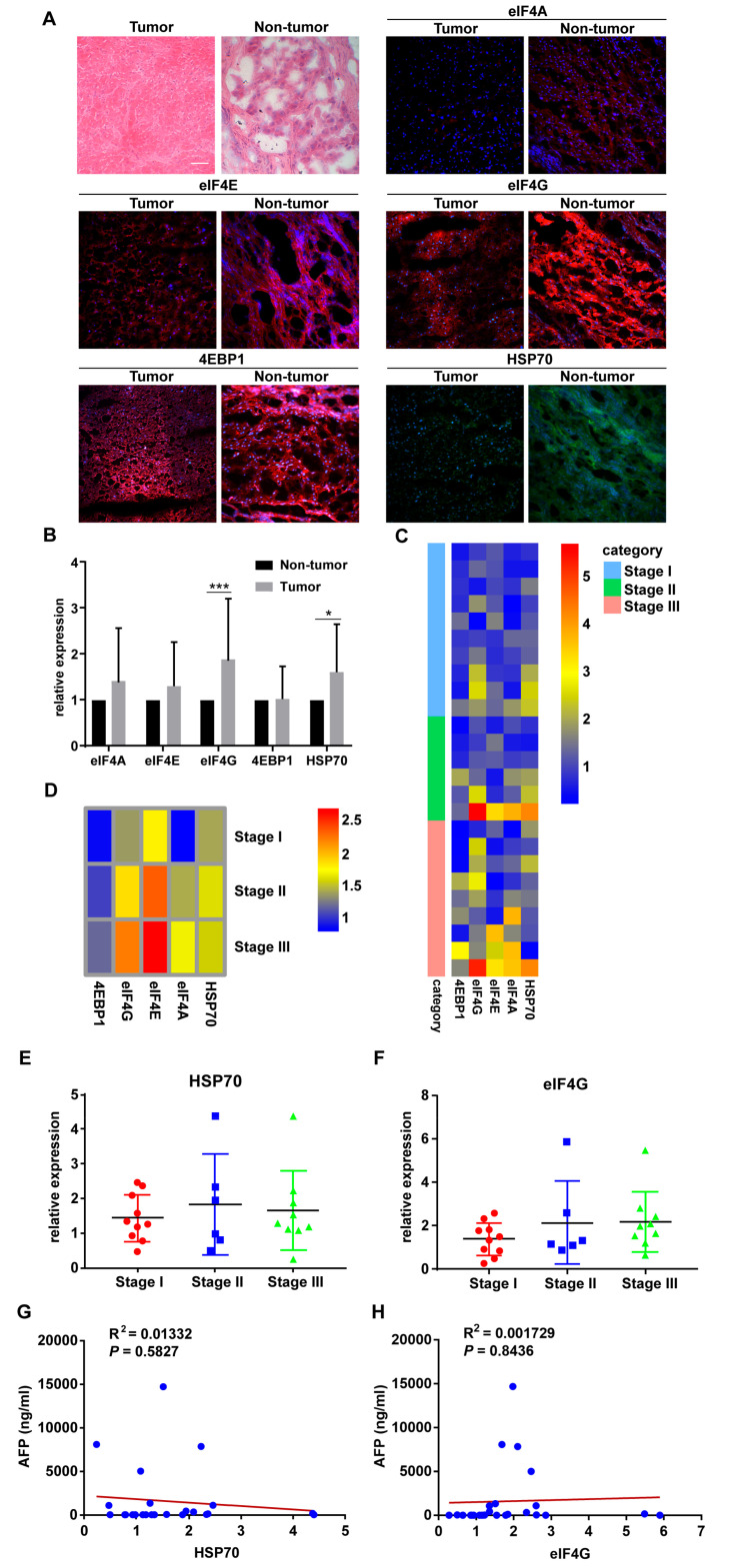
 should be replaced with the following version:

**Figure 1:**
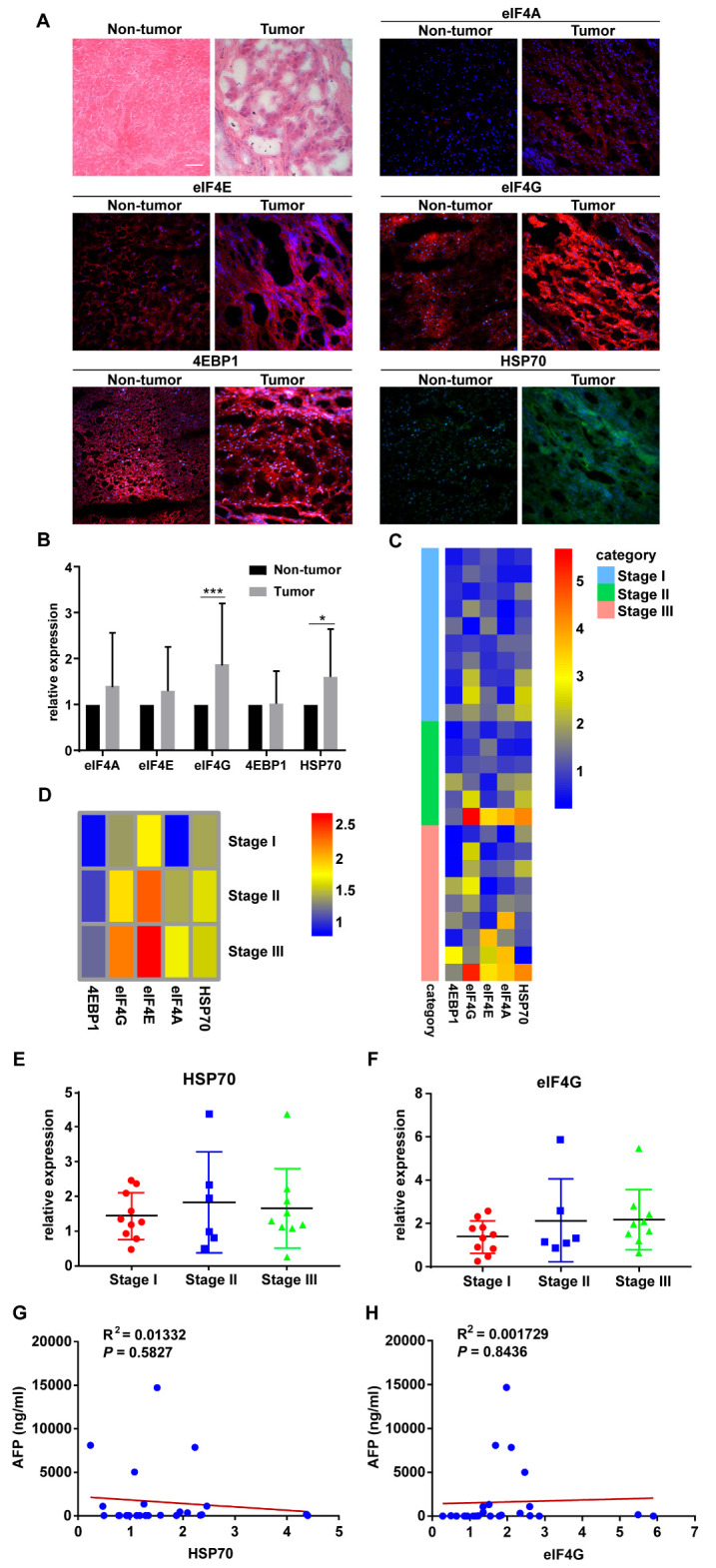
HSP70 and eIF4G expression are significantly higher in hepatocellular carcinoma (HCC) tumor specimens. (**A**) Representative pictures of H&E and IF staining. Magnification, 20×; Scale bar, 50μm. (**B**) The protein levels of HSP70 and eIF4G in HCC tumor specimens were significantly higher than those of adjacent non-tumor specimens. (**C**) Heatmap showing the relative protein expression of eIF4A, eIF4E, eIF4G, 4EBP1, and HSP70 in all HCC patients tissue samples based on IF staining results. (**D**) Heatmap showing the average relative protein expression of eIF4A, eIF4E, eIF4G, 4EBP1, and HSP70 in all patients in each TNM stage. (**E**,**F**) Protein expression of HSP70 and eIF4G in HCC patients displayed according to the TNM stage. (**G**,**H**) The scatter plot of correlation between the AFP level and the protein expression of HSP70 and eIF4G. * *p* < 0.05, *** *p* < 0.001 compared with the adjacent non-tumor groups.

The authors state that this correction does not modify the scientific results of the study. The authors would like to apologize for any inconvenience caused to the readers by these changes. The manuscript will be updated, and the original will remain online on the article webpage.
